# Evaluating Computerised Assessment of Motor Imitation (CAMI) for identifying autism-specific difficulties not observed for attention-deficit hyperactivity disorder or neurotypical development

**DOI:** 10.1192/bjp.2024.235

**Published:** 2026-01

**Authors:** Romila Santra, Carolina Pacheco, Deana Crocetti, René Vidal, Stewart H. Mostofsky, Bahar Tunçgenç

**Affiliations:** Center for Neurodevelopmental and Imaging Research, Kennedy Krieger Institute, Baltimore, Maryland, USA; Mathematical Institute for Data Science, Johns Hopkins University, Maryland, USA; and Department of Biomedical Engineering, Johns Hopkins University, Maryland, USA; School of Engineering and Applied Science, University of Pennsylvania, Pennsylvania, USA; and Perelman School of Medicine, University of Pennsylvania, Pennsylvania, USA; Center for Neurodevelopmental and Imaging Research, Kennedy Krieger Institute, Baltimore, Maryland, USA; Department of Neurology, Johns Hopkins University School of Medicine, Maryland, USA; and Department of Psychiatry and Behavioral Sciences, Johns Hopkins University School of Medicine, Maryland, USA; Department of Psychology, Nottingham Trent University, UK; and Institute of Human Sciences, University of Oxford, UK

**Keywords:** Autism spectrum disorders, attention-deficit hyperactivity disorders, diagnostic medicine, social functioning, neurodevelopmental disorders

## Abstract

**Background:**

Reliable and specific biomarkers that can distinguish autism spectrum disorders (ASDs) from commonly co-occurring attention-deficit/hyperactivity disorder (ADHD) are lacking, causing misses and delays in diagnosis, and reducing access to interventions and quality of life.

**Aims:**

To examine whether an innovative, brief (1-min), videogame method called Computerised Assessment of Motor Imitation (CAMI), can identify ASD-specific imitation differences compared with neurotypical children and children with ADHD.

**Method:**

This cross-sectional study used CAMI alongside standardised parent-report (Social Responsiveness Scale, Second Edition) and observational measures of autism (Autism Diagnostic Observation Schedule-Second Edition; ADOS-2), ADHD (Conners) and motor ability (Physical and Neurological Examination for Soft Signs). The sample comprised 183 children aged 7–13 years, with ADHD (without ASD), with ASD (with and without ADHD) and who were neurotypical.

**Results:**

Regardless of co-occurring ADHD, children with ASD showed poorer CAMI performance than neurotypical children (*P* < 0.0001; adjusted *R^2^* = 0.28), whereas children with ADHD and neurotypical children showed similar CAMI performance. Receiver operating curve and support vector machine analyses showed that CAMI distinguishes ASD from both neurotypical children (80% true positive rate) and children with ADHD (70% true positive rate), with a high success rate significantly above chance. Among children with ASD, poor CAMI performance was associated with increased autism traits, particularly ADOS-2 measures of social affect and restricted and repetitive behaviours (adjusted *R^2^* = 0.23), but not with ADHD traits or motor ability.

**Conclusions:**

Four levels of analyses confirm that poor imitation measured by the low-cost and scalable CAMI method specifically distinguishes ASD not only from neurotypical development, but also from commonly co-occurring ADHD.

Autism spectrum disorder (ASD) is an early-developing condition affecting one in 36 children in the USA and one in 100 children globally.^1,2^ Early diagnosis of ASD enables access to effective interventions and improvement in quality of life;^3^ however, we lack cost-effective, specific and reliable biomarkers and tests^4,5^ that can distinguish children with ASD not just from neurotypical children, but also those with commonly co-occurring conditions such as attention-deficit hyperactivity disorder (ADHD) (50–70% co-occurrence^6^), resulting in a clouded clinical picture causing missed or delayed ASD diagnoses.^7^ Motor imitation performance is a promising autism-specific biomarker, with imitation being critical for social learning and relationships,^8^ and poor imitation widely reported in children with ASD compared with neurotypical children.^9,10^ Prior studies further suggest an ASD-specific motor profile, as compared with ADHD, in tasks such as ball-catching and dyspraxia,^11,12^ with an over-reliance on proprioceptive, as opposed to visual, feedback in children with ASD.^13,14^ Going forward, identifying the unique motor profiles specific to ASD versus ADHD will be crucial for precision medicine approaches to diagnosis and guiding interventions.

Establishing motor imitation as a biomarker for autism requires robust methods with high sensitivity and specificity. For decades, motor imitation assessments relied on human observation coding, which has limited reliability, precision and scalability because it is time-consuming and requires multiple highly trained observers. Addressing these challenges, we recently developed the Computerised Assessment of Motor Imitation (CAMI), a brief, engaging videogame-like task that assesses imitation performance using computer vision methods without attaching any wearables to the children and virtually without any human input for data processing. Our published findings have shown that for children aged 7–13 years, the CAMI distinguishes ASD diagnosis from neurotypical development more robustly than the traditional, human observation coding methods,^15,16^ with the CAMI approaching 90% accuracy. These findings are initial steps toward establishing the CAMI as a reliable assessment method that can capture the autism phenotype without relying on language. However, before the CAMI can be enrolled more widely in younger age groups and more severely affected ASD populations, we need to advance its specificity by demonstrating that it identifies autism-specific imitation difficulties as different from other co-occurring conditions where atypical motor profiles are observed, such as ADHD, developmental coordination disorder or developmental language disorder.^17–19^

## Current study

To this end, in this study, we tested four groups of children: ADHD without ASD, ASD with co-occurring ADHD (ASD + ADHD), ASD without ADHD (ASD only) and neurotypical. Based on previous research, we hypothesised that children with ASD would show poorer imitation (i.e. lower CAMI scores) than children with ADHD and neurotypical children, whereas the latter two would not significantly differ from each other. In addition, we examined dimensional metrics of motor ability, and ASD and ADHD symptom severity, hypothesising that in children with ASD, CAMI performance would be associated with core ASD traits rather than with ADHD traits or motor ability. Finally, we conducted receiver operating curve (ROC) and support vector machine (SVM) analyses to examine how successfully CAMI scores alone could distinguish children with ASD from children with ADHD and neurotypical children, by calculating its specificity and sensitivity.

## Method

### Participants

Participants were 183 children aged 7–13 years old: 35 with ADHD (without ASD), 63 with ASD and co-occurring ADHD (ASD + ADHD), 21 with ASD without ADHD (ASD only) and 65 neurotypical children (see Table 1 for detailed sample characteristics). One additional child from the ASD-only group was tested but excluded from analysis because their CAMI score was more than three times above the interquartile range of the ASD sample. ADHD subtypes within the ADHD group were 12 inattentive, 22 combined and one not otherwise specified. ADHD subtypes within the ASD + ADHD group were 25 inattentive, six hyperactive/impulsive, 31 combined and one not otherwise specified.

**Table 1:** Sample characteristics of each group

	ADHD	ASD + ADHD	ASD only	Neurotypical	Significance (Bonferroni corrected *t*-test)
*n*	35	63	20	65	
Age, years, mean (s.d.)	10.06 (1.49)	10.62 (1.36)	9.95 (1.03)	10.33 (1.34)	Not significant
Gender, female/male	14/21	8/55	2/18	18/47	*χ*^2^(3) = 12.22, *P* = 0.01
Ethnicity, White/other	28/7	48/15	12/8	49/16	Not significant
WISC-V GAI, mean (s.d.)	108.03 (17.00) (*n* = 33)	102.92 (17.14) (*n* = 60)	104.90 (17.24) (*n* = 20)	114.98 (12.69) (*n* = 60)	Neurotypical versus ASD + ADHD: *P* = 0.0003
PANESS total, mean (s.d.)	26.32 (8.45) (*n* = 19)	36.37 (12.45) (*n* = 38)	39.07 (10.34) (*n* = 14)	22.83 (9.48) (*n* = 58)	ADHD versus ASD + ADHD: *P* = 0.005 ADHD versus ASD only: *P* = 0.004 ASD + ADHD versus neurotypical: *P* < 0.0001 ASD only versus neurotypical: *P* < 0.0001
ADOS-2 total, mean (s.d.)	–	12.72 (3.92)	14.72 (5.20)	–	Not significant
SRS-2 total, mean (s.d.)	59.83 (12.12) (*n* = 6)	74.92 (8.96) (*n* = 53)	69.42 (6.27) (*n* = 19)	45.78 (7.37) (*n* = 50)	ASD + ADHD versus neurotypical: *P* < 0.0001 ASD only versus neurotypical: *P* < 0.0001 ASD + ADHD versus ASD only: *P* = 0.03
Conners inattention, mean (s.d.)	71.57 (11.11) (*n* = 30)	72.68 (10.90) (*n* = 47)	61.00 (11.12) (*n* = 17)	45.56 (7.37) (*n* = 57)	ADHD versus ASD only: *P* = 0.003 ADHD versus neurotypical: *P* < 0.0001 ASD + ADHD versus neurotypical: *P* < 0.0001 ASD only versus neurotypical: *P* < 0.0001 ASD + ADHD versus ASD only: *P* = 0.0003
Conners hyperactivity/impulsivity, mean (s.d.)	68.37 (13.17) (*n* = 30)	71.51 (13.65) (*n* = 47)	61.24 (9.58) (*n* = 17)	46.91 (8.02) (*n* = 57)	ADHD versus neurotypical: *P* < 0.0001 ASD + ADHD versus neurotypical: *P* < 0.0001 ASD only versus neurotypical: *P* < 0.0001 ASD + ADHD versus ASD only: *P* = 0.009
CAMI, mean (s.d.)	0.42 (0.22) (*n* = 35)	0.29 (0.16) (*n* = 63)	0.29 (0.13) (*n* = 20)	0.51 (0.20) (*n* = 65)	ADHD versus ASD + ADHD: *P* = 0.007 ASD + ADHD versus neurotypical: *P* < 0.0001 ASD only versus neurotypical: *P* < 0.0001

ADHD, attention-deficit hyperactivity disorder; ASD, autism spectrum disorder; WISC-V GAI, Wechsler Intelligence Scale for Children General Ability Index, Fifth Edition; PANESS, Physical and Neurological Examination for Soft Signs; ADOS-2 total, T-scores of the Autism Diagnostic Observation Schedule, Second Edition; SRS-2 total, T-scores of the Social Responsiveness Scale, Second Edition; Conners, Conners ADHD Index; CAMI, Computerised Assessment of Motor Imitation.

Testing took place as part of a 2-day visit at the Center for Neurodevelopmental and Imaging Research, Kennedy Krieger Institute, Baltimore, Maryland, USA. All procedures complied with the ethical standards of the national and institutional committees on human experimentation and with the Helsinki Declaration of 1975, as revised in 2008. All procedures involving human participants were approved by the Johns Hopkins University School of Medicine Institutional Review Board (approval number IRB00269589). Parents of all participants provided written informed consent, and the children provided verbal assent before data collection. Parents were reimbursed $50 for participation.

ASD diagnosis was confirmed with the clinician-administered Autism Diagnostic Observation Schedule-Second Edition, Module 3 (ADOS-2);^20^ and ADHD diagnosis was confirmed with the Kiddie Schedule for Affective Disorders and Schizophrenia^21^ and Conners,^22^ according to DSM-5 criteria. All participants had a full-scale IQ of ≥70, as measured by the Wechsler Intelligence Scale for Children, Fifth Edition (WISC-V),^23^ to ensure they could follow the instructions.

### Measures and procedure

Children completed an imitation task, from which their CAMI scores were obtained. The children's parents filled out the Social Responsiveness Scale-Second Edition (SRS-2)^24^ and the Conners-3 or Conners-4^22^ rating scale, which were used as measures of the children's autism and ADHD traits, respectively. In addition, children completed the Physical and Neurological Examination for Soft Signs (PANESS)^25^ as a measure of their motor ability, and the children in the ASD group were administered the ADOS-2.^20^

#### CAMI

Imitation was assessed over two trials, each comprising a 1-min, highly engaging game, in which children were asked to copy the dance-like, whole-body movements of a video avatar while standing. The children's movements were recorded with two sensorless Kinect Xbox cameras located directly opposite and behind them. Following the raw data processing steps and the CAMI algorithm described in previous studies,^15,16^ we calculated the children's imitation score for each trial. Identifying the most important joints for each move automatically in a data-driven manner, CAMI calculates an imitation score per trial that varies between 0 (no imitation at all) and 1 (perfect imitation, i.e. a well-trained researcher's imitation of the avatar). The two trials featured different movement sequences, and the scores in the two trials were significantly correlated with each other (*r*(179) = 0.76, *P* < 0.0001). Hence, CAMI scores from the two trials were averaged to obtain a composite CAMI score per participant that ranged from 0 to 1, with higher scores indicating better imitation performance.

#### ADOS-2

The ADOS-2^20^ is a semi-structured, activity-based standardised assessment of autism-associated traits. There are five available modules, each designed for a different age group and expressive language level. All participants in this study were administered Module 3. Observational coding by the test administrator yields a total score and two subscale scores: social affect and restrictive and repetitive behaviours. Total comparison scores range from 1 to 10, where 1–2 indicates minimal to no evidence of ASD, 3–4 indicates a low level of ASD symptoms, 5–7 indicates a moderate level of ASD symptoms and 8–10 indicates a high level of ASD symptoms. All ASD participants in this study had a total score range of 6–27.

#### SRS-2

The SRS-2^24^ is a 65-item, parent-report questionnaire intended to quantify the social communication difficulties experienced in ASD. The scale yields a total score, as well as five subscales: social awareness, social cognition, social communication, social motivation, and restricted interests and repetitive behaviour. Overall T-scores were used in analyses, with higher scores indicating more autistic traits. According to the scale guidelines, scores <60 indicate no social communication difficulties, scores of 60–75 indicate mild-to-moderate difficulties and scores >75 indicate severe difficulties.

#### Conners

The Conners-3 and Conners-4 are parent-report rating scales used to assess ADHD and other co-occurring disorders in children aged 6–18 years, using 108 items and 114 items, respectively. DSM-IV-TR (DSM-V for Conners-4) subscales include inattention and hyperactivity/impulsivity, as well as three validity scales. T-scores from the DSM-based ADHD Index inattention and hyperactivity/impulsivity subscales were used in this study. In both subscales, T-scores have a mean of 50 and s.d. of 10, with higher scores indicating more difficulties with inattention or hyperactivity/impulsivity. According to scale guidelines, scores of 40–59 are considered within neurotypical range, scores of 60–64 are considered high average/slightly elevated, scores of 65–69 are considered elevated and scores >70 are considered very elevated, indicating many more concerns than reported in neurotypical populations. Participants in the ADHD and ASD + ADHD in this study had inattentiveness score range of 42–90, and hyperactivity/impulsivity score range of 38–90.

#### PANESS

The PANESS^25^ is a standardised behavioural examination administered to assess motor abilities of children aged 6–17 years. PANESS assesses motor domains, including gait, balance and timed movements, and returns both subscale scores for each domain and an overall summed motor ability (PANESS total) score. Previous literature shows poorer motor skills as measured by PANESS in children with ASD and children with ADHD.^26–28^ Scores are scaled based on age, gender and handedness norms, with higher scores indicating poorer motor ability. In this study, PANESS total scores were used.

### Data analysis

All analyses were conducted in RStudio for Windows version 2023.06.0+21 (RStudio Team, PBC, Boston, MA; see http://www.rstudio.com/), using lme4^29^ and tidyr^30^ packages. To examine whether children with ASD would show poorer imitation than children with ADHD and neurotypical children, we used a linear regression model, with the predictor variable being the four diagnostic groups (ADHD versus ASD + ADHD versus ASD only versus neurotypical), the outcome variable being the children's imitation performance as measured by CAMI score, and WISC-V General Ability Index and participant gender as the covariate, given the observed diagnostic group differences in the latter two variables. Bonferroni-corrected pairwise *t*-test was conducted to examine which diagnostic groups differed from each other, and Cohen's *d*-statistic was calculated to indicate effect sizes of diagnostic group differences.

To examine whether CAMI performance is associated with core ASD traits rather than with ADHD traits or motor ability, we used a linear regression model within the ASD group (ASD + ADHD and ASD-only combined), with the predictor variables being dimensional metrics of autism traits (SRS-2 and ADOS-2 total scores), ADHD traits (Conners inattention and hyperactivity/impulsivity scores) and motor ability (PANESS scores), and the outcome variable being CAMI scores. To explore whether the associations between the dimensional metrics and CAMI held similarly in other populations, linear regression models were conducted within the ADHD and neurotypical groups as well. Because of the high amount of missing data for the SRS-2 and PANESS from the ADHD group, and the ADOS-2 not being administered to the ADHD and neurotypical groups (see Table 1), these dimensional metrics were excluded from the predictors of the respective models. Multicollinearity between predictors was satisfactory for all models (generalised variance inflation factor (GVIF) range: 1.01–1.59). In addition, different dimensions of autism traits were explored for children with ASD, with the predictor variable being ADOS-2 subscale scores (i.e. social affect and restricted and repetitive behaviours), the outcome variable being CAMI scores and the ASD subgroups (i.e. ASD + ADHD versus ASD only) being the moderator variable. For all models, adjusted *R*^2^-values are provided to indicate effect sizes by accounting for the number of predictors in regressions models.

To examine the diagnostic classification ability of CAMI scores, we trained SVM classifiers by using three-fold cross-validation (i.e. two folds for training, one fold for test). The folds were balanced on age, gender and IQ. SVM with linear kernel was used as a classifier, with the misclassification cost being inversely proportional to the number of samples per class to address the class imbalance. To improve interpretability of the results, we used a very simple model consisting of a linear SVM with only two learnable parameters, which means that the classification performance using our sample size versus an infinite amount of samples would be negligible, i.e. <0.2%.^31^ In addition, we computed the ROC of the children's CAMI score across trials to confirm that the classification ability of CAMI is not specific to machine learning. The ROC curve is generated by plotting the true positive rate versus false positive rate for 500 classification thresholds uniformly distributed in the [0 1] range. The area under the curve (AUC) quantifies the diagnostic classification ability of the scores, with larger values indicating better classification.

## Results

### Differences in imitation performance across diagnostic groups

**Fig. 1:**
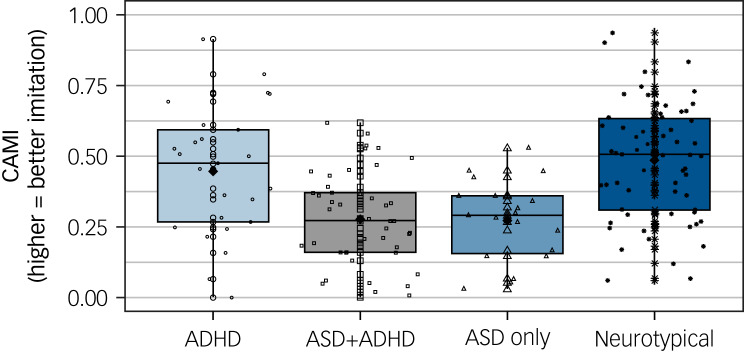
Imitation performance across diagnostic groups (*n*_ADHD_ = 35, *n*_ASD_ _+_ _ADHD_ = 63, *n*_ASD-only_ = 20, *n*_neurotypical_ = 65). Horizontal lines inside the box plots show the median, black diamonds show the mean and the dots show individual data points within each diagnostic group. ADHD, attention-deficit hyperactivity disorder; ASD, autism spectrum disorder; CAMI, Computerised Assessment of Motor Imitation. *P < 0.01, **P < 0.0001.

Linear regression model examining diagnostic group differences in imitation scores was significant (*F*(5,167) = 14.32, *P* < 0.0001, adjusted *R^2^* = 0.28), with diagnosis (*F*(3,167) = 18.01, *P* < 0.0001; see Fig. 1), WISC-V General Ability Index scores (*F*(1,167) = 5.74, *P* = 0.02) and participant gender (*F*(1,167) = 11.83, *P* = 0.001) significantly predicting CAMI scores. Of note, linear regressions with the WISC-V General Ability Index scores and participant gender as predictors of CAMI scores within each diagnostic group revealed no significant associations for WISC-V General Ability Index scores in either group (all *P* > 0.05), but a significant gender effect within the neurotypical group only (*F*(1,57) = 8.24, *P* = 0.006), such that neurotypical girls (mean 0.62, s.d. = 0.21) had higher CAMI scores than neurotypical boys (mean 0.46, s.d. = 0.21).

Bonferroni-corrected pairwise comparisons examining the diagnostic group differences revealed that children with ADHD performed similarly to neurotypical children (*P* = 0.13, *d* = 0.44). In contrast, children in both the ASD + ADHD (*P* < 0.0001, *d* = 1.20) and ASD-only (*P* < 0.0001, *d* = 1.13) groups had significantly lower imitation scores than neurotypical children. Children in the ASD + ADHD group had significantly lower imitation scores than children with ADHD (*P* = 0.007, *d* = 0.71), whereas children in the ASD-only group performed similarly to children with ADHD (*P* = 0.12, *d* = 0.64). These results largely support our hypothesis in that imitation difficulties as measured by CAMI are observed specifically in ASD, and not in ADHD, as compared with neurotypical populations. However, contrary to our predictions, the results did not show a significant difference between ADHD and ASD-only groups, which, given the similarity in the distributions of the two ASD groups, likely stems from the low sample size in the ASD-only group (see Discussion for more detailed discussion of this point).

### Associations of imitation with ASD, ADHD and motor features

**Fig. 2:**
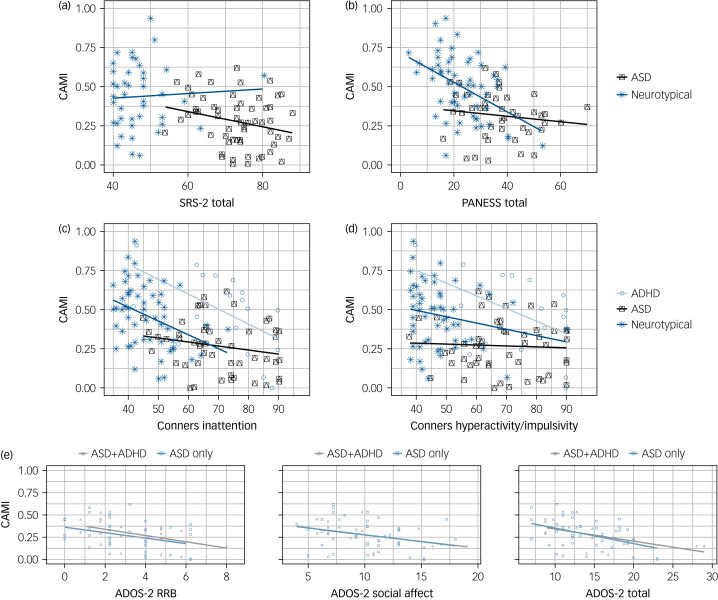
Associations of imitation with ASD traits, ADHD traits and motor ability. Scatter plots showing associations of the CAMI with (a) SRS-2 total scores, (b) PANESS total scores, (c) Conners inattention scores, (b) Conners-3 hyperactivity/impulsivity scores and (e) ADOS-2 restricted and repetitive behaviours and social affect subscales and total scores. Colour codes for the groups are as follows: light blue for ADHD (*n* = 35), black for combined ASD (*n* = 84), dark blue for neurotypical (*n* = 65), light blue for ASD + ADHD (*n* = 63) and dark blue for ASD only (*n* = 20). ADHD, attention-deficit hyperactivity disorder; ASD, autism spectrum disorder; ADOS-2, Autism Diagnostic Observation Schedule-Second Edition; CAMI, Computerised Assessment of Motor Imitation; PANESS, Physical and Neurological Examination for Soft Signs; RRB, restricted and repetitive behaviours; SRS-2, Social Responsiveness Scale-Second Edition.

Within the ASD group, the model assessing how dimensional metrics of ASD, ADHD and motor features predict CAMI was significant (*F*(5,29) = 2.99, *P* = 0.03, adjusted *R^2^* = 0.23). Among the predictors, only ADOS-2 total scores significantly predicted CAMI (*F*(1,29) = 5.90, *P* = 0.02), with poorer imitation being observed as autism traits increased. Additional linear models examining each ADOS-2 subscale further showed that higher scores in both the social affect (*F*(3,74) = 3.30, *P* = 0.03, adjusted *R^2^* = 0.08) and the restricted and repetitive behaviours (*F*(3,74) = 5.40, *P* = 0.002, adjusted *R^2^* = 0.15) subscales significantly predicted lower CAMI scores. These findings support our secondary hypothesis in that imitation performance in ASD is related with core autism traits, not ADHD traits or motor ability. Examination of how these associations manifest within the ADHD and neurotypical groups revealed that the dimensional metrics model was not significant within ADHD (*F*(2,27) = 0.41, *P* = 0.67, adjusted *R^2^* = −0.04), but it was significant within the neurotypical group (*F*(4,37) = 8.08, *P* < 0.0001, adjusted *R^2^* = 0.41). For neurotypical children, Conners inattention (*F*1,37) = 10.43, *P* = 0.003) and PANESS (*F*(1,37) = 21.75, *P* < 0.0001) scores significantly predicted CAMI scores, with higher attention and motor ability being associated with better CAMI performance. Associations of CAMI with SRS-2, PANESS and Conners metrics for the ADHD, ASD and neurotypical groups are shown in Fig. 2(a)–2(d), and associations with ADOS-2 subscale and total scores for the ASD subgroups are shown in Fig. 2(e). Altogether, these findings support our secondary hypothesis by showing that in ASD, imitation is associated specifically with autism traits, not ADHD traits or motor ability, whereas in neurotypical children, imitation is associated with attentional and motor abilities.

### Diagnostic classification ability of the CAMI

**Fig. 3:**
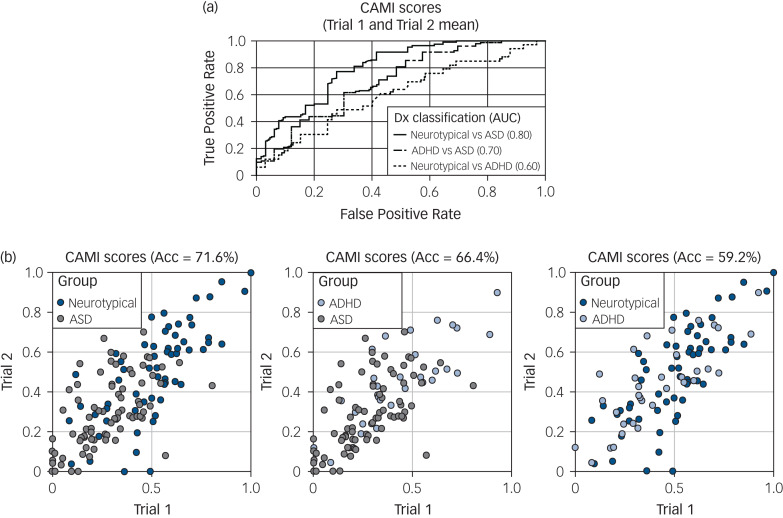
Diagnostic classification ability of the CAMI confirmed by two complementary methods. (a) Receiver operating curve reveals high true positive rates (area under the curve) for distinguishing children with ASD (*n* = 84) from neurotypical children (*n* = 65) is 80%, and from children with ADHD (*n* = 33) is 70%. (b) Three-fold cross-validated support vector machine results show high classification accuracy for distinguishing children with ASD from neurotypical children (72%) and children with ADHD (66%). ADHD, attention-deficit hyperactivity disorder; ASD, autism spectrum disorder; AUC, area under the curve; CAMI, Computerised Assessment of Motor Imitation.

The ROC analysis revealed that the true positive rate, as indicated by the AUC, was 80% for the CAMI's ability to distinguish children with ASD from neurotypical children and 70% for its ability to distinguish children with ASD from children with ADHD, whereas the AUC for distinguishing children with ADHD from neurotypical children was 60% (Fig. 3(a)). Chi-squared analysis with Yates correction for each of these comparisons revealed that although children with ASD were correctly classified significantly more than chance as compared with both neurotypical children (*χ^2^*(1) = 15.15, *P* < 0.0001) and children with ADHD (*χ^2^*(1) = 6.41, *P* = 0.01), the classification of children with ADHD from neurotypical children was not significantly different from chance levels (*χ^2^*(1) *=* *0.25, P* *=* 0.62). These results align with SVM analyses, which revealed that CAMI scores alone could distinguish children with ASD from neurotypical children with 71.6% accuracy, children with ASD from children with ADHD with 66.4% accuracy, and children with ADHD from neurotypical children with 59.2% accuracy (Fig. 3(b)). Cumulatively, these findings evidence CAMI's specificity as an autism marker – CAMI can distinguish children with ASD from neurotypical children and children with ADHD with a success rate significantly above chance. In contrast, children with ADHD and neurotypical children show similar motor profiles in terms of their CAMI performance.

## Discussion

This study demonstrated that motor imitation difficulties, as measured with the automated, brief (1-min) and engaging CAMI method, are specifically observed in ASD, but not in ADHD, as compared with neurotypical children. These results largely, but not wholly, support our hypothesis, because although the ADHD group's CAMI scores were higher than the ASD + ADHD group, they were not significantly different from the ASD-only group. For children with ASD, imitation was closely related to core autism traits. In contrast, for neurotypical children, imitation was associated with inattention and motor ability. These findings support our secondary hypothesis that imitation difficulties in ASD are associated with autism traits, rather than with ADHD traits or motor ability. Finally, both ROC and SVM analyses revealed that the CAMI distinguishes children with ASD from neurotypical children and children with ADHD with a high success rate that is significantly above chance, whereas the CAMI performance of children with ADHD is indistinguishable from that of neurotypical children.

The key contribution of this study is laying the groundwork for establishing the specificity of the CAMI as a biomarker of ASD. Regardless of co-occurring ADHD, children with an ASD diagnosis had significantly poorer CAMI performance as compared with neurotypical children. Although the CAMI performance of children with ADHD did not significantly differ from children in the ASD-only group, this is likely a by-product of low power resulting from the small sample size. Indeed, the CAMI performance of children with ADHD was significantly different from children in the ASD with co-occurring ADHD (i.e. ASD + ADHD) group, a group which had three times the sample size of the ASD-only group, with very similar mean and s.d. values. The specificity of the CAMI to ASD was demonstrated using four different analytic approaches: diagnostic group comparisons, nuanced dimensional metrics, ROC analyses and SVM machine learning methods. Converging results from these different types of analyses support the use of CAMI as a reliable and highly scalable method for detecting ASD based on motor imitation differences. Of note, our machine learning analysis shows that CAMI can distinguish children with ASD from both neurotypical children and children with ADHD with significant success, pointing to its strong potential for diagnostic precision.

Although the CAMI is currently a research tool whose reliability, validity and some degree of specificity has been established, it has significant potential in clinical practice. Before clinical use, however, further study is needed in terms of its longevity, specificity to autism and utility with younger and more affected (e.g. non-verbal, intellectually disabled) children. Our study provides evidence that not inattention or poor motor ability, but rather autistic traits, and social communication skills in particular, are associated with imitation in ASD. Future longitudinal research can elucidate the direction of this association, i.e. whether it is impaired social communication that leads to imitation difficulties or early emerging imitation difficulties that affect social communication function throughout the lifespan. The latter possibility is in line with previous literature, which has found that imitation skills of infants with ASD predict language development and play skills in later childhood,^32^ but not necessarily motor skills.^33^ Of note, and in line with theoretical models of motor imitation,^34,35^ better attentional and motor skills were associated with better imitation performance for neurotypical children. To understand these mechanisms in more detail, future research can not only utilise CAMI at earlier ages and longitudinally, but also use imaging techniques during CAMI to examine visual-motor integration mechanisms underlying imitation performance.

We utilised both parent-report and clinician-observed measures of autistic traits. Although the parent-report measure (SRS-2) did not correlate with imitation performance, the clinician-observed measure (ADOS-2) did. This discrepancy may speak to a tendency for parents to report their child to be less affected in communication and social interaction domains as compared with clinicians.^36^ It may also be that direct behavioural observation via the ADOS-2 provides richer, more comprehensive information than does memory-based reportage in SRS-2. Whatever the reason for the discrepancy, these findings indicate that using measures from different reporters is critical to gain a more complete understanding of ASD.

### Limitations

Given the heterogeneity of ASD and ADHD presentation, sample sizes of *n* = 20 for the ASD-only group and *n* = 35 for the ADHD group are relatively small. This sample size limitation may have accounted for our finding of statistically non-significant differences between the ADHD and ASD-only groups, calling for replication of these findings with larger sample sizes. In addition, missing data on several metrics in the ADHD group did not allow examination of autism traits and motor ability metrics within ADHD. It remains to be understood whether imitation performance in ADHD is associated with the same factors as neurotypical children. Additionally, given that imitation skills develop in early childhood,^8^ future studies should adapt CAMI for use with younger children. Doing so will increase the utility of CAMI as a diagnostic and intervention tool, given that the average age of ASD diagnosis is 10 years old^37^ and earlier diagnosis enables more effective interventions. Further, our ASD sample primarily included children of relatively high intellectual ability (i.e. an IQ score of >70). Future iterations of the task should aim to simplify instructions so they are accessible to those with intellectual disability, which would more wholly represent the wide intellectual range within the ASD population.

In conclusion, by using multiple levels of analyses – diagnostic group comparisons, dimensional associations, classification analyses using ROC and SVM – this study provides evidence that, as compared with neurotypical children, motor imitation difficulties, measured using the highly reliable and scalable CAMI method, are specific to ASD, and not shared with the frequently co-occurring neurodevelopmental condition ADHD. Imitation skills appear to have divergent pathways in neurotypical children and children with ADHD and ASD: although imitation is associated with attention and motor skills in neurotypical children, for children with ASD, imitation is related to core autism traits. Finally, ROC and SVM analyses showed strong agreement and further confirm that imitation ability, as measured by CAMI alone, distinguishes children with ASD from both neurotypical children (80% true positive rate) and children with ADHD (70% true positive rate) with high accuracy, whereas the CAMI performances of children with ADHD and neurotypical children are nearly indistinguishable (60% true positive rate). These findings provide the basis for establishing the CAMI as a biomarker specific to ASD.

## Data Availability

In line with the funder's requirements, anonymised data will be made available at the National Database for Autism Research upon publication (http://ndar.nih.gov/). Research materials and analysis scripts will be made available on the Open Science Framework page of the corresponding author, B.T.
